# The Book World of Medicine and Science

**Published:** 1900-12-08

**Authors:** 


### The Book World of Medicine and Science.

Consumption: Its Nature and Treatment. By W. H. Spencer, M.D., M.R.C.P. Pp. 82. (London: H. J. Glaisher. 1900.)

Like many others, Dr. Spencer has a plan for the curing of consumption. But this plan is not in this little book set forth, nor are we made acquainted with histories of its successful prosecution. But we gather that it involves the inhalation of antiseptics, the inhalation being practised in accordance with the laws of diffusion of gases. But we of the profession can hardly complain that the details of this plan are not now divulged to us, inasmuch as the author (who is a Consulting Physician to the Bristol Infirmary) frankly states that his aim is to instruct the intelligent and educated public. To this end he has written some account of consumption from which we gather that in his own opinion consumption is not always tubercular, and that it is better not to call consumption "tuberculosis." Dr. Spencer's attitude towards what he calls the "bacillary theory of consumption" is mystifying and mysterious. He admits "the transference of and the evil influence of bacillary products" as an element in the case; but, having it from Koch himself that caseous matter in which no bacilli can be detected is infective, argues the existence of some other "matters," transferable and transferred. But the phenomena of spore formation are completely ignored by Dr. Spencer. Apart from the promulgation of these and other curious pathological errors, there is little of interest in the book. There is much repetition of dreary pomposities such as these:—"Medical art could never heal disease," "To this end, cure, and to the help of nature, medical art can do great things," and so forth. In fact, we can see no useful purpose in this book. It can only irritate and grieve medical men, and will hopelessly befog any layman who may read it. If Dr. Spencer has benefited cases of consumption by his special methods, let him bring the facts before the profession. But it is deplorable that a gentleman of Dr. Spencer's professional standing should write such a book as this, for the "instruction of the public," without previously submitting his methods to the judgment of his fellow-physicians.

Children of Scorn and Medical Papers. By Lady Cook (*née* Tennessee Claflin). London: The National Union Publishing Company.

This is a most disagreeable book in every sense. The first part is largely devoted to a protest against the lifelong hardship which is imposed upon those borne out of wedlock and incidentally against the treatment meted out by society to their unfortunate mothers. One need not, however, dip far into its pages to discover that the real protest is against those social conditions which lead so many men and women to remain unmarried during that period of their lives when the sexual desire is at the strongest. This is avowedly the object of the second part of the book, which the authoress by a strange perversion of language is pleased to call "medical" papers. Her idea is that "maternity is the birthright of every healthy woman, and paternity of every healthy man." But since under present arrangements some have too many children and some too few, "it becomes the moral as well as the natural duty of everyone to practise some sort of effectual restraint in the bearing and begetting of children." In old countries where poverty exists large families are an unmitigated evil, "and since the desire for children is a natural instinct, and all have a natural right to their production, it becomes a social wrong to exercise that right in such a manner as to exclude the same right in others." One need hardly say that a lady who sets up such standards of rights and wrongs goes far. We are told that "everyone who gives birth to more than two prevents at least one other woman from enjoying the physical advantages and wholesome influences and pleasures of maternity." "And since it is the easiest thing in the world to regulate the number of one's children and still to have a proper amount of sexual intercourse, all these evils might be reduced to the smallest dimensions by the general knowledge of a few physiological facts and a determination to profit by them." "Under such circumstances the poorest could marry, and all might marry early." The writer of this wretched trash takes care not to enter into details, but we may say at once that all her notions are founded upon the fundamental error of believing that the exercise of the sexual function is essential and that "health of body and mind demands obedience to this instinct." Which is nonsense.

The Proper Discipline to be Observed by Married People in Regard to Conjugal Intercourse.

Counsel for Young and Unmarried Men respecting Chastity. Issued on high medical authority, with episcopal sanction. (London: John Bale, Sons and Danielsson. Price 2d. post free. Issued also in packets.)

These leaflets will we think be found useful by medical practitioners. It is not always easy to formulate one's advice on this subject, even when it is asked for, while it is often difficult to give it when it is not requested. Many patients are shy and nervous in regard to all such topics, and it may often happen that the giving of a printed leaflet in the course of a consultation may be a more satisfactory proceeding than dealing by word of mouth with a subject the mere speaking of which is to many an offence. Both these leaflets show a tendency to refer to Scripture, which we are afraid will with many, in this worldly age, detract from rather than add to their authority, so great is the desire in all things medical to be up to date. Nevertheless the advice given is eminently sane, and may be most properly accepted as authoritative.

How We Escaped from Pretoria. By Captain Aylmer Haldane, D.S.O., 2nd Batt. Gordon Highlanders. (Edinburgh and London: William Blackwood and Sons. 1900. Price 1s.)

Those who imagine that at the present period of the world's history adventure is a thing of the past had better read this little book which describes in simple language, albeit with a certain dash of humour, the hardships, the trials, and the hairbreadth escapes which were undergone by Captain Haldane in making his way out of his place of captivity in Pretoria. The dodges and devices which he and his companion had to make use of, the patience they displayed, and the manner in which at times, notwithstanding all the odds against them, they got through, while at other times with everything apparently in their favour their schemes came to nothing, are explained in full and make the book interesting from end to end.

